# Tetramethylpyrazine Ameliorates High Glucose-Induced Endothelial Dysfunction by Increasing Mitochondrial Biogenesis

**DOI:** 10.1371/journal.pone.0088243

**Published:** 2014-02-05

**Authors:** Qiong Xu, Pu Xia, Xi Li, Wei Wang, Zhenqi Liu, Xin Gao

**Affiliations:** 1 Department of Endocrinology and Metabolism, Zhongshan Hospital, Fudan University, Shanghai, China; 2 The Key Laboratory of Molecular Medicine, Ministry of Education, Department of Biochemistry and Molecular Biology, Fudan University Shanghai Medical College, Shanghai, China; 3 Division of Endocrinology and Metabolism, Department of Medicine, University of Virginia Health System, Charlottesville, Virginia, United States of America; Idaho State University, United States of America

## Abstract

Tetramethylpyrazine (TMP) is an active compound isolated from a Chinese herbal prescription that is widely used in traditional Chinese medicine for the treatment of inflammatory and cardiovascular diseases. We have previously reported that TMP acts as a potent antioxidant protecting endothelial cells against high glucose-induced damages. However, the molecular mechanism responsible for the antioxidant effect of TMP remains to be elucidated. In this study, we show that TMP increases nitric oxide production in endothelial cells and promotes endothelium-dependent relaxation in rate aortic rings. The antioxidant effect of TMP appears attributable to its ability to activate the mitochondrial biogenesis, as reflected in an up-regulation of complex III and amelioration of mitochondrial membrane potential. Furthermore, TMP is able to reverse high glucose-induced suppression of SIRT1 and the biogenesis-related factors, including PGC-1α, NRF1 and TFAM, suggesting a new molecular mechanism underlying the protective effect of TMP on the endothelium.

## Introduction

Nitric oxide (NO) produced by endothelium cells is of vital importance for maintaining the integrity and normal function of the endothelium. The bioactivity of NO includes relaxing smooth muscle cells, inhibiting adhesion molecule expression, preventing leukocyte adhesion and migration, platelet aggregation and vascular inflammation. Endothelial dysfunction, featured as a reduced NO production and/or utilization, is the earliest event in the development of cardiovascular diseases, including atherosclerosis, hypertension and diabetic cardiovascular disease [1]–[3]. Accumulation of reactive oxygen species (ROS) in endothelial cells is the main factor causing endothelial dysfunction [4]–[6]. Thus anti-oxidative stress has emerged an important strategy to prevent and treat diabetic complication and atherosclerosis [7]–[8]. However, clinical trials using the traditional antioxidants such as vitamin C, vitamin E and folic acid in patients with cardiovascular diseases showed neutral or even negative results [9]–[11]. One reason for these unfavorable results is that these antioxidants are not able to accumulate in mitochondria to effectively scavenge the superoxide anions. Thus, developing new types of antioxidants that have more potency, higher bioactivity, and more site-specificity, is of significant importance for the prevention and treatment of diabetes and atherosclerosis [8], [12].

Many studies have demonstrated that some natural products of traditional Chinese medicine (TCM), such as flavonoids, are promising antioxidants and strong candidates for the treatment of endothelial dysfunction [13]–[15]. As reported in our previous study, the herbal remedy Qiong Huo Yi Hao (QHYH) that consists of several herbals based on the “clearing heat and detoxifying” principle of TCM, exhibits a potent effect to scavenge superoxide anions in high glucose-treated endothelial cells [16]. Tetramethylpyrazine (TMP), a chemical compound isolated from QHYH, was demonstrated being the strongest one among all isolated products of QHYH in promoting NO generation and reducing ROS formation in high glucose-treated endothelial cells [17]. However, the molecular mechanism underlying the antioxidant effect of TMP remains to be elucidated.

Peroxisome proliferator-activated receptor gamma coactivator-1α (PGC-1α), a key transcriptional factor involved in the first step of mitochondrial biogenesis, plays a crucial role in preventing of oxidative stress and endothelial cell dysfunction [18]. SIRT1, a mammalian homologue of Sir2, has been shown to promote PGC-1α deacetylation, thereby regulating stress responses, apoptosis, and cellular senescence [19]. Interestingly, TMP was shown in our previous study to alleviate palmitate-induced mitochondrial ROS production through the PGC-1α mediated mitochondrial biogenesis pathway in C2C12 cells [17]. Therefore it is a temptation to validate whether TMP could alleviate high glucose-induced mitochondrial dysfunction through the SIRT1-dependent pathway.

In this study, we report that TMP increases NO production by endothelial cells and promotes endothelium-dependent relaxation in rate aortic rings. TMP treatment resulted in a significant up-regulation of complex III and ameliorated mitochondrial membrane potential in endothelial cells. Furthermore, TMP is able to reverse high glucose-induced suppression of SIRT1 and the biogenesis-related factors, including PGC-1α, NRF1 and TFAM. These findings thus suggest that the antioxidant TMP acts on the mitochondrial biogenesis pathway, which may account for the protective effect of TMP on the endothelium.

## Materials and Methods

### Regents and materials

Male Sprague-Dawley (SD) rats were purchased from Sina-British Sippr/Bk Lab Animal Ltd. (Shanghai, China). Tetramethylpyrazine (TMP), Crocin, Ferulic acid and Chlorogenic acid, acetylcholine, phenylephrine (PE) and D-glucose were purchased from Sigma-Aldrich Co. (St Louis, Missouri, USA). Dulbecco's modified Eagle's medium (DMEM) supplemented with 5.6 mmol/L glucose was obtained from Hclone (Logan, UT, USA). DMEM without red phenol, fetal bovine serum (FBS) and Trizol were purchased from Invitrogen (Carlsbad, CA, USA). CM-H_2_DCFDA, DAF-FM diacetate and MitoSOX™ Red mitochondrial superoxide indicators were obtained from Invitrogen (Carlsbad, CA, USA). JC-1 indicator was bought from Molecular Probes (Eugene, OR, USA). Antibodies including anti-complex III, anti-PGC-1α, anti-SIRT1 and anti-β-actin were purchased from Proteintech (ProteinTech Group, Chicago, IL, USA). Primers for PGC-1α, NRF-1, TFAM, SIRT1 and 18s were provided by Sangon Biotech CO. Ltd. (Shanghai).

### Cell Culture

Human umbilical venular endothelial cells (HUVEC) were isolated from freshly obtained human umbilical cords by 0.1% collagenase and 0.1% trypsin digestion [20] and maintained in endothelial cell medium (ECM; Sciencell Research Laboratories Inc., CA, USA). Endothelial cells were characterized by their non-overlapping cobblestone morphology with CD31 positive staining. Experiments were performed on HUVEC at 3–6 generations. The use of human umbilical cords for the study was approved by the Human Research Ethics Committee of Zhongshan Hospital, and was conducted according to the principles of the Declaration of Helsinki. Written informed consent was obtained from all subjects. The murine brain microvascular cell line bEnd.3 was purchased from American Type Culture Collection. Cells were cultured in DMEM containing 5.6 mmol/L glucose, 100 U/mL penicillin, 0.1 mg/mL streptomycin and 10% FBS at 37°C in a humidified atmosphere of 5% CO_2_. The medium was changed every 2 days.

### Preparation of isolated rat aortic rings

All animal experiments were approved by the Animal Research Ethics Committee of Zhongshan Hospital, and conform to the Guide for the Care and Use of Laboratory Animals published by US National Institutes of Health (NIH publication No. 85-23, revised 1996). Male SD rats were anesthetized with intraperitoneal injection of 1 g/kg ethyl urethane. The thoracic aorta were isolated, followed by the removal of adhering connective tissues, and then cut in 3–4 mm ring segments [21]. The rings were suspended between two stainless steel stirrups in an organ bath filled with 10 ml Krebs solution at 37°C. The composition of Krebs solution was (in mmol): NaCl, 118; KCl, 4.7; CaCl_2_, 2.5; MgSO_4_, 1.18; KH_2_PO4, 1.08; NaHCO_3_, 25; Glucose, 11; and bubbled with 95%O_2_ +5% CO_2_ (PH 7.35–7.45) [22]. Organ bath was changed with Krebs solution every 15 mins. The upper stirrup was connected to a force transducer (Alcott Biotech, Shanghai, China) and the lower one fixed to the bottom of the organ bath.

### Assessment of endothelium integrity of rat aortic rings

The integrity of endothelium was assessed by the relaxation response to acetylcholine (10^−5^ mol/L) in rings pre-contracted with phenylephrine (10^−6^ mol/L). For some rings, the endothelium was disrupted by rubbing the internal surface with a polyethylene tube. The aortic rings with the relaxant response >80% were considered as E+ and the rings with no relaxant response were considered as E−.

### Treatment of aortic rings with TMP

After loaded under a tension of 2 g and equilibrated for 60 mins in Krebs solution, the rings were challenged with 60 mmol/L KCl until a stable contraction was achieved, and later were washed three times with Krebs solution every 10 mins. Then these E+ and E− aortic rings were challenged with 10^−6^ mol/L phenylephrine again to achieve the maximum contraction. Finally, TMP was added into organ bath to obtain the concentration-dependent response curves from the treated aortic rings.

### Measurement of NO content

HUVEC and bEnd.3 cells were seeded in a 96-well plate at density of 8000 cells per well and cultured for 1 d and then treated with 5.6 mmol/L glucose (normal glucose, Control), 30 mmol/L glucose (high glucose, HG) or 30 mmol/L glucose + 30 µmol/l TMP for 48 hrs, respectively. The working concentration of TMP was determined in accordance to our previous study [17]. NO production was measured using DAF-FM diacetate indicator according to the method described previously [23]. Briefly, the treated cells were incubated with 5 µmol/L DAF-FM diacetate for 25 mins and then washed twice by phosphate-buffered saline (PBS). The fluorescence signal of NO was visualized by fluorescent microscopy (Olympus BX50) and measured by flexstation 3 multi-mode microplate reader (Molecular Device) at an excitation wavelength of 495 nm and an emission wavelength of 515 nm. NO production was represented by fold changes in the fluorescence signals compared with the controls.

### Determination of ROS production

Cells were seeded in a 96-well plate at the intensity of 8000 cells per well. After attached for 1 d, cells were cultured in DMEM supplemented with 1% FBS and grouped as the following treatments: (i) 5.6 mmol/L glucose (normal glucose, Control), (ii) 30 mmol/L glucose (high glucose, HG) and (iii) 30 mmol/L glucose +30 µmol/L TMP. After the treatment for 48 hrs, CM-H_2_DCFDA indicator was used to determine ROS production as described previously [24]. The treated cells were incubated with 5 µmol/l CM-H_2_DCFDA for 25 mins. After washed twice with PBS, the ROS fluorescence signal was visualized by fluorescence microscopy and measured by flexstation 3 multi-mode microplate reader at an excitation wavelength of 485 nm and emission wavelength of 530 nm. ROS production was represented as fold changes in fluorescence compared with the normal glucose-treated controls.

### Assay for superoxide anion

MitoSOX™ Red was used to determine mitochondrial superoxide anion concentration in endothelial cells. Cells were treated as the following four groups: (i) 5.6 mmol/L glucose (normal glucose, Control), (ii) 30 mmol/L glucose (high glucose, HG), (iii) 5.6 mmol/L glucose + 30 µmol/l TMP, and (iv) 30 mmol/L glucose +30 µmol/L TMP for 48 hrs. MitoSOX™ Red is reported being selectively targeted to intracellular mitochondria where the reagent is oxidized by superoxide and exhibits red fluorescence [25]. The red fluorescence signal was visualized by fluorescence microscopy.

### Measurement of the mitochondrial membrane potential

The mitochondrial membrane potential was determined by using the mitochondrial dye JC-1 (5,5′,6,6′-tetrachloro-1,1′,3,3′-tetraethylbenzimidazolocarbocyanine iodide). JC-1 exhibits potential-dependent accumulation in mitochondria, indicated by a fluorescence emission shift from green (∼530 nm) to red (∼590 nm). Accordingly, mitochondrial depolarization is indicated by a decrease in the red/green fluorescence intensity ratio. Briefly, endothelial cells were cultured in a 24-well plate with different treatments as described above, then incubated with 10 µg/ml of JC-1 for 30 mins at 37°C, and scanned with flexstation 3 multi-mode microplate reader. The fluorescence ratio (590 to 530 nm) was used for quantitative analysis [26].

### RNA isolation and RT-PCR

Total RNA was isolated using the TRIzol reagent (Invitrogen) according to the manufacturer's instruction. cDNA was synthesized using the RevertAid™ First Strand cDNA Synthesis Kit (Fermentas, Glen Burnie, MD). Real-time quantitative PCRs were performed with 2× PCR Master Mix (Power SYBR Green; Applied Biosystems, Foster City, CA) on PRISM7900 (ABI, Applied Biosystems, FosterCity, CA). The primer sequences for PGC-1, NRF-1, TFAM and SIRT1 are shown in table 1. The threshold cycles (Ct) for each gene were determined in triplicate experiments, and the relative mRNA quantity was calculated using the comparative Ct method.

**Table 1:** Primers used for Real-time PCR.

Gene	Forward Primer (5′→3′)	Reverse Primer (5′→3′)
**Mouse**		
SIRT1	AGTTCCAGCCGTCTCTGTGT	CTCCACGAACAGCTTCACAA
PGC-1α	ATGTGTCGCCTTCTTGCTCT	ATCTACTGCCTGGGGACCTT
NRF-1	TTGGAACAGCAGTGGCAAGA	CTCACTTGCTGATGTATTTACTTCCAT
TFAM	GTCGCATCCCCTCGTCTATC	GCTGGAAAAACACTTCGGAATAC
18s-mouse	CGCCGCTAGAGGTGAAATTCT	CATTCTTGGCAAATGCTTTCG
**Human**		
SIRT1	TGGCAAAGGAGCAGATTAGTAGG	CTGCCACAAGAACTAGAGGATAAGA
PGC-1α	AGCTTTGGCTTTACGGAATACCA	CCACAGGATAAGTCACCGAGGA
NRF-1	GGCACTGTCTCACTTATCCAGGTT	CAGCCACGGCAGAATAATTCA
TFAM	CCGAGGTGGTTTTCATCTGT	GCTGAACGAGGTCTTTTTGG
18s-human	GTAACCCGTTGAACCCCATT	CCATCCAATCGGTAGTAGCG

### Western blot assay

The treated cells as described above were lysed in lysis buffer containing 2% SDS, 10 mM DTT, 50 mM Tris-HCl, pH 6.8, 10% glycerol, 0.002% bromophenol blue, and 1× protease inhibitor mixture. Protein concentrations were determined by the BCA-100 Protein Quantitative Analysis Kit. Equal amounts of proteins were separated by 10% SDS-PAGE, which were transferred onto polyvinylidene fluoride membranes (Millipore, Billerica, MA), blocked by 5% skim milk in Tris-buffered saline containing 0.1% Tween 20 for 1 h and then washed for 3 times. The proteins were immunoblotted with primary antibodies against complex III, PGC-1α and SIRT1, respectively, for overnight at 4°C. After washed for 3 times, the blots were incubated with secondary antibodies (anti-rabbit or anti-mouse IgG) for 1 h at room temperature. Then, the protein bands were visualized by chemiluminescence (ECL) detection reagents and quantified by Fujifilm Las-3000 Luminescent Image Analyzer.

### Statistical analysis

All data were expressed as SD±SEM, and the number of samples referred as n. One-way ANOVA (GraphPad, Version 5) was used to determine statistical significance, employing Bonferonni corrections for multiple comparisons. A *p* value <0.05 was considered to be significant.

## Results

### Effect of TMP on endothelium-dependent vasodilatation of aortic rings

**Figure 1:**
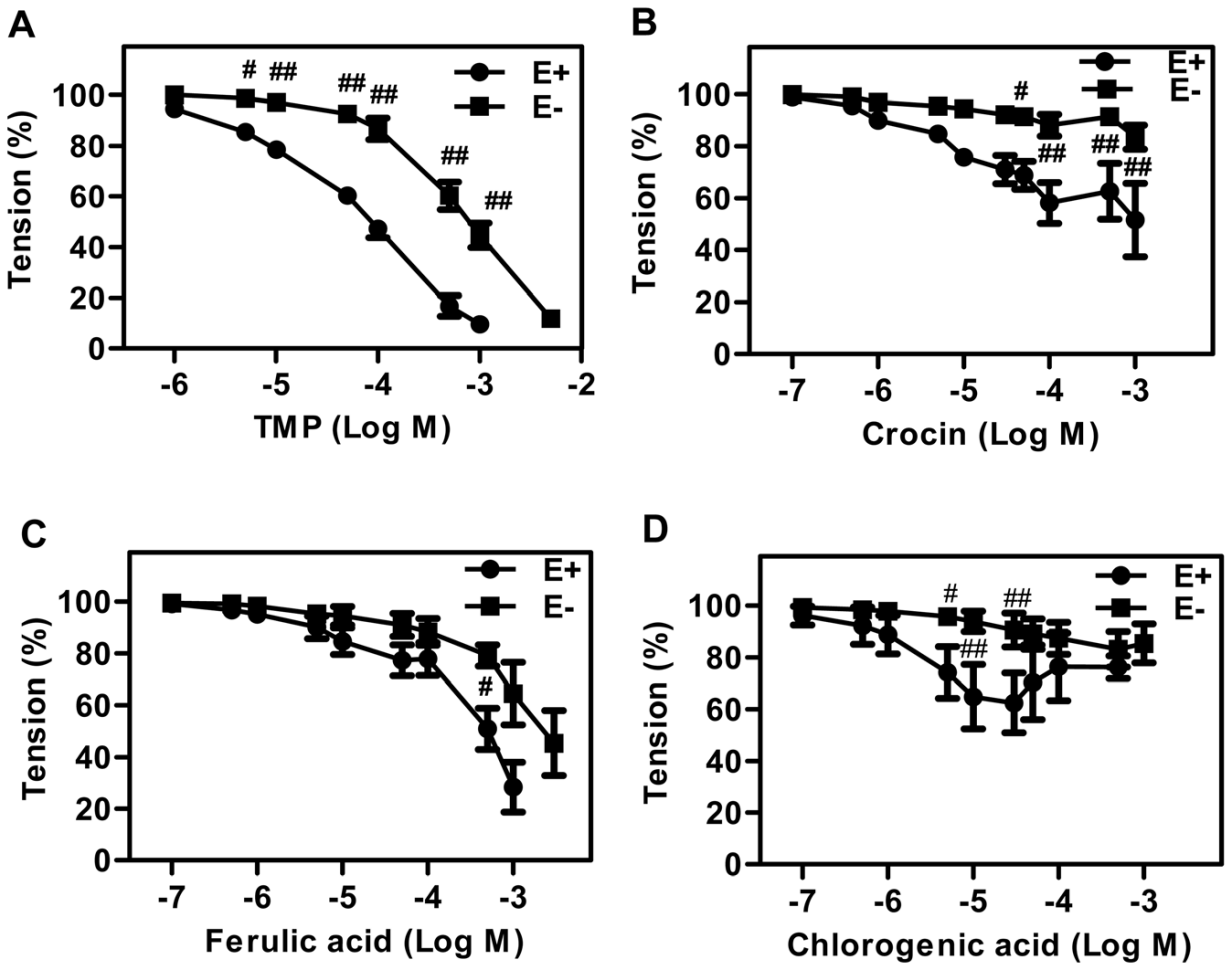
Vasorelaxant effects of TMP on rat aortic rings. The freshly isolated rat aortic rings were treated with (**A**) TMP, (**B**) crocin, (**C**) ferulic acid and (**D**) chlorogenic acid at increasing concentrations. After the treatments, both endothelium-intact (E+) and endothelium-denuded (E−) ring relaxation were measured as described in the Methods. Data are mean ± SEM (n = 4–7). ^##^ *P*<0.01 vs. E+; ^#^ *P*<0.05 vs. E+.

Rat aortic rings were used to detect the vasodilatation effects of TMP. As sown in Figure 1A, TMP treatment induced an evident relaxation of either endothelium-stripped (E−) or endothelium-intact (E+) rat aortic rings in a concentration-dependent manner. At the concentrations ranging from 5 µmol/L to 1 mmol/L, TMP-induced relaxation of endothelium-intact aortic rings was significantly larger than that of endothelium-stripped aortic rings (*P*<0.05), suggesting that TMP actions chiefly on the endothelial-dependent vasodilation (Emax 90.36%). Serving as controls for the TMP treatment, other three chemical compounds isolated from the herbal remedy QHYH including crocin, chlorogenic acid and ferulic acid were used. Although both crocin and chlorogenic acid exhibited a relaxant effect on endothelium-intact aortic rings (Figure 1B and 1D), these effects were only observed within a small concentration range, and the maximum relaxant effects were weaker than that of TMP (Emax 41.27%, Emax 39.44% vs. Emax 90.36%). In addition, ferulic acid treatment resulted in a relaxation of both endothelium-stripped and endothelium-intact aortic rings. However, there were no significant differences between the E− and E+ groups (Figure 1C), indicating the relaxant effect of ferulic acid was endothelium independent.

### Effect of TMP on high glucose-induced suppression of NO in bEnd.3 cells

**Figure 2:**
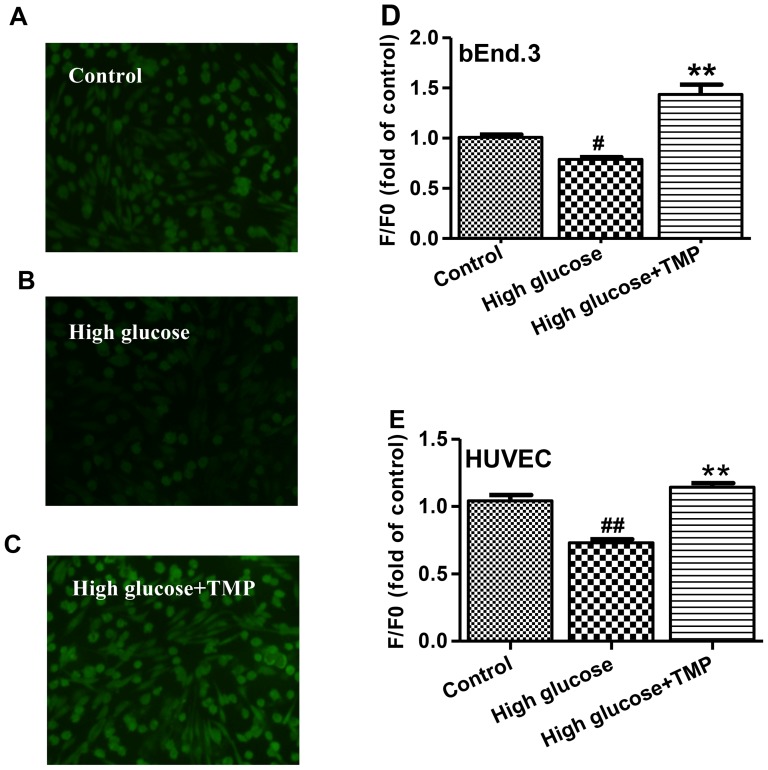
TMP increased NO production in endothelial cells exposed to high glucose. DAF-FM indicator was used to detect NO levels in HUVEC treated with (**A**) 5.6 mmol/L glucose (Control), (**B**) High glucose (30 mmol/L) and (**C**) High glucose+TMP (30 µmol/L). The treated cells were photographed by fluorescence microscopy (**A–C**). (**D**) bEnd.3 and (**E**) HUVEC were treated as indicated for 48 hrs, and NO production was measured using flexstation3 and quantified with the changes in fluorescence intensity. “F0” represents fluorescence of Control group and “F” depicts fluorescence of other treated groups. Data are mean ± SEM (n = 4). ^#^ *P*<0.05 vs. Control; ^##^ *P*<0.01 vs. Control; ^*^ *P*<0.01 vs. High glucose.

DAF-FM diacetate indicator was used to detect NO production. Exposure of bEnd.3 cells to 30 mmol/l glucose for 48 hrs resulted in a significant reduction in NO production, compared with normal glucose treatment. Remarkably, treated with 30 µmol/L TMP, the high glucose-induced suppression of NO production was completely restored (Figure 2). However, as we reported previously, treatment with TMP alone had no effect on NO production under normal glucose conditions [17]. The results demonstrate a potent effect of TMP in prevention of bEnd.3 cells from high glucose-induced NO reduction.

### Antioxidant effect of TMP on high glucose-treated bEnd.3 cells

**Figure 3:**
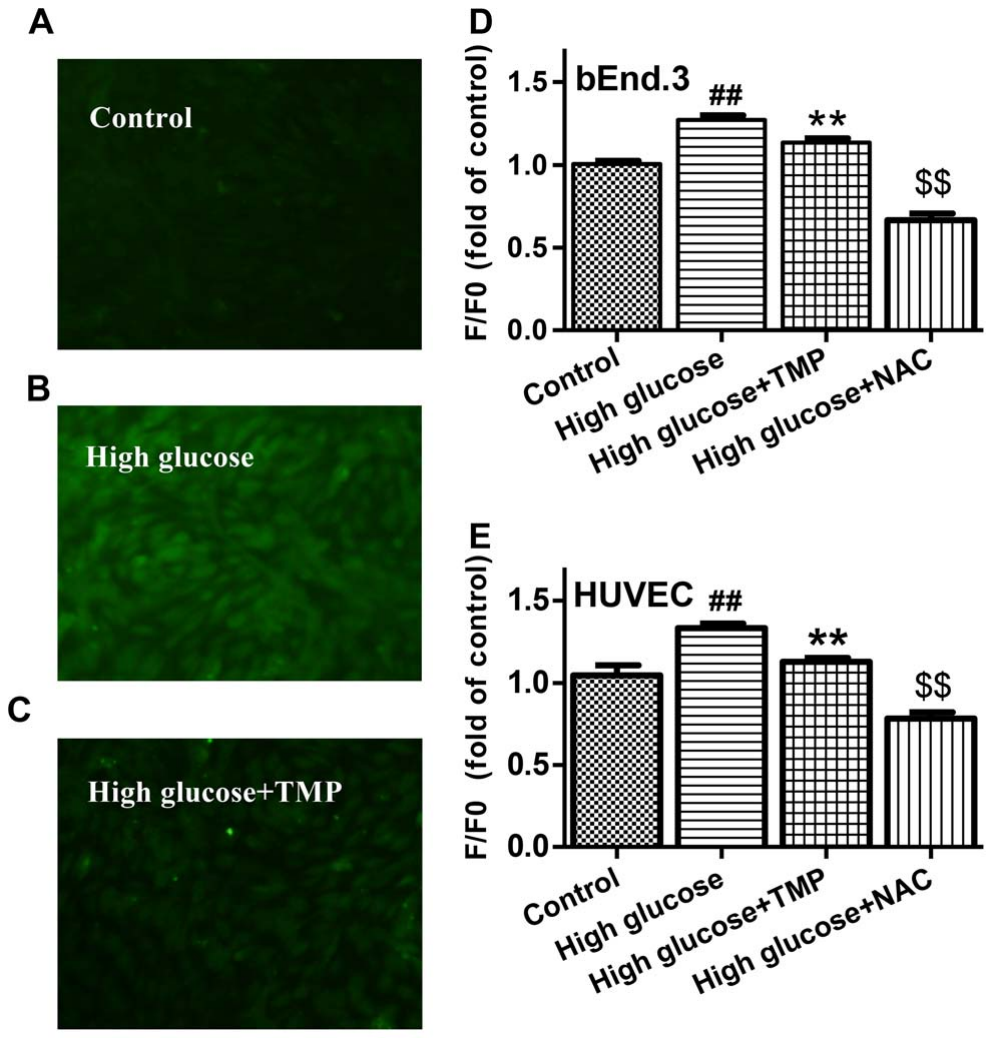
TMP decreased ROS generation in high glucose-treated endothelial cells. CM-H_2_DCFDA indicator was used to detect ROS levels in HUVEC treated with (**A**) 5.6 mmol/L glucose (Control), (**B**) High glucose (30 mmol/L) and (**C**) High glucose+TMP (30 µmol/L). (**D**) bEnd.3 and (**E**) HUVEC were treated as indicated for 48 hrs, and ROS production was measured using flexstation3 and quantified with the changes in fluorescence intensity. “F0” represents fluorescence of Control group and “F” depicts fluorescence of other treated groups. Data are mean ± SEM (n = 4). ^#^ *P*<0.05 vs. Control; ^##^ *P*<0.01 vs. Control; ^**^ *P*<0.01 and ^$$^ *P*<0.01 vs. High glucose.

**Figure 4:**
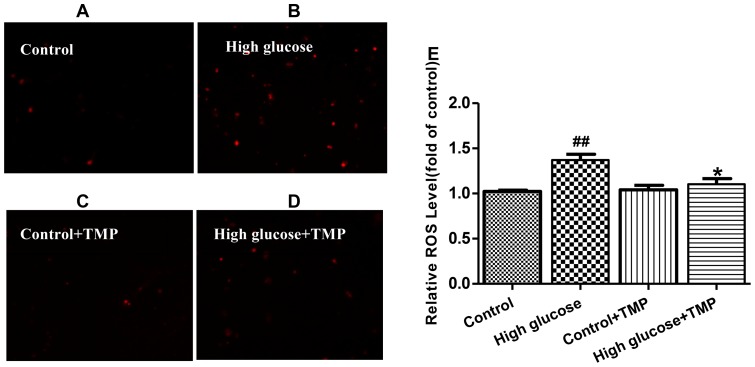
TMP decreased superoxide anions in high glucose-treated endothelial cells. Superoxide anions were detected in HUVEC treated with (**A**) 5.6 mmol/L glucose (Control), (**B**) High glucose (30 mmol/L), (**C**) Control+ TMP (30 µmol/L) and (**D**) High glucose+TMP (30 µmol/L) by using MitoSOX™ Red. Fluorescence intensity was recorded by fluorescence microscopy, and the quantification data are shown (**E**). Data are mean ± SEM (n = 4). ^##^ *P*<0.01 vs. Control; ^*^ *P*<0.05 vs. High glucose.

As expected, HUVEC and bEnd.3 cells exposed to 30 mmol/L glucose for 48 hrs resulted in a significant increase in ROS generation (Figure 3). The high glucose -induced ROS production was markedly inhibited by the treatment with 30 µmol/l TMP, suggesting an antioxidant effect of TMP in endothelial cells. The antioxidant effect of TMP was further confirmed by the assays of mitochondrial superoxide anion using the MitoSOX™ Red indicators in live cells. The MitoSOX™ Red reagent can be oxidated by superoxide in mitochondria, displaying red fluorescence. As shown in Figure 4, the red fluorescence in high glucose-treated endothelial cells was stronger than that in the control cells, indicating more mitochondrial superoxide produced. Treatment with TMP reduced the fluorescence in endothelium cells exposed to high glucose, demonstrating the effect of TMP to reduce high glucose-induced superoxide anion generation in mitochondria (Figure 4).

### TMP alleviated mitochondrial dysfunction induced by high glucose

**Figure 5:**
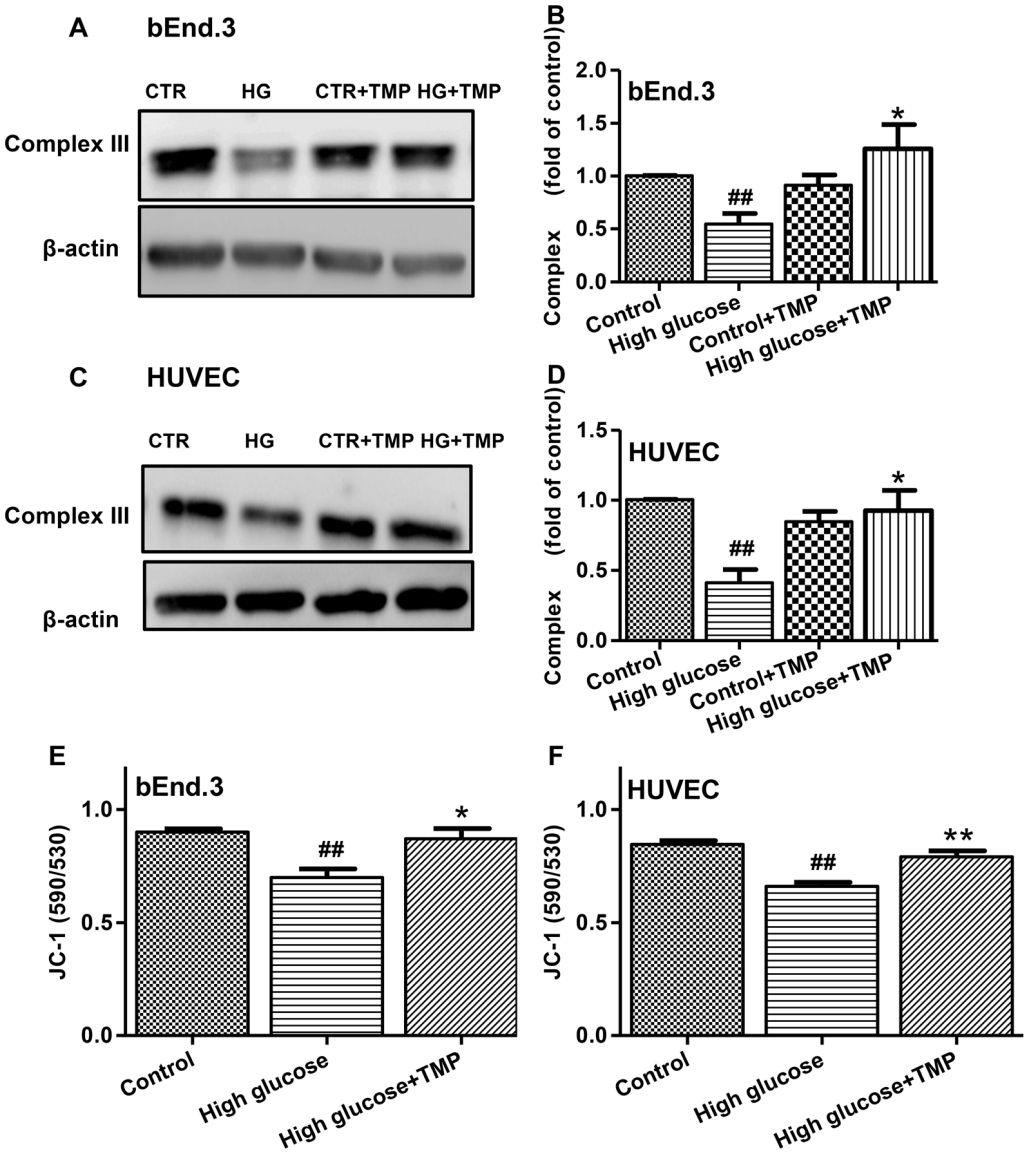
The protective effects of TMP against mitochondrial dysfunction in endothelial cells. The bEnd.3 and HUVEC cells were treated with Control (5.6 mmol/L glucose, CTR), High glucose (30 mmol/L, HG), Control+ TMP (30 µmol/L) and High glucose+TMP (30 µmol/L) for 48 hrs. Levels of complex III expression were analyzed by Western blotting in bEnd.3 (**A** and **B**) and HUVEC cells (**C** and **D**). Representative blots (**A** and **C**) and quantification data (**B** and **D**) are shown. Mitochondrial potential were determined by JC-1 indicator in the treated bEnd.3 (**E**) and HUVEC cells (**F**). Data are mean ± SEM (n = 4). ^#^ *P*<0.05 vs. Control group; ^##^ *P*<0.01 vs. Control; ^*^ *P*<0.05 vs. High glucose; ^**^ *P*<0.01 vs. High glucose.

The accumulation of superoxide anions in high glucose-treated endothelial cells could lead to mitochondrial dysfunction [27]–[28]. The function of mitochondrial complex III, one of the most import proteins in the respiratory chain, is often impaired in the early stage of mitochondrial dysfunction [27]. Accordingly, complex III protein expression was chosen as an indicator to evaluate mitochondrial function. The results show that high glucose incubation decreases complex III protein expression in endothelial cells compared with the controls. Significantly, TMP treatment reversed the reduction of complex III in endothelial cells exposed to high glucose (Figure 5A–D). Moreover, high glucose-induced decreases in the mitochondrial membrane potential were reversed by TMP treatment in endothelial cells. Together, these results suggest that TMP is able to alleviate mitochondrial dysfunction induced by high glucose.

### TMP enhanced mitochondrial biogenesis through up-regulation of PGC-1α expression

**Figure 6:**
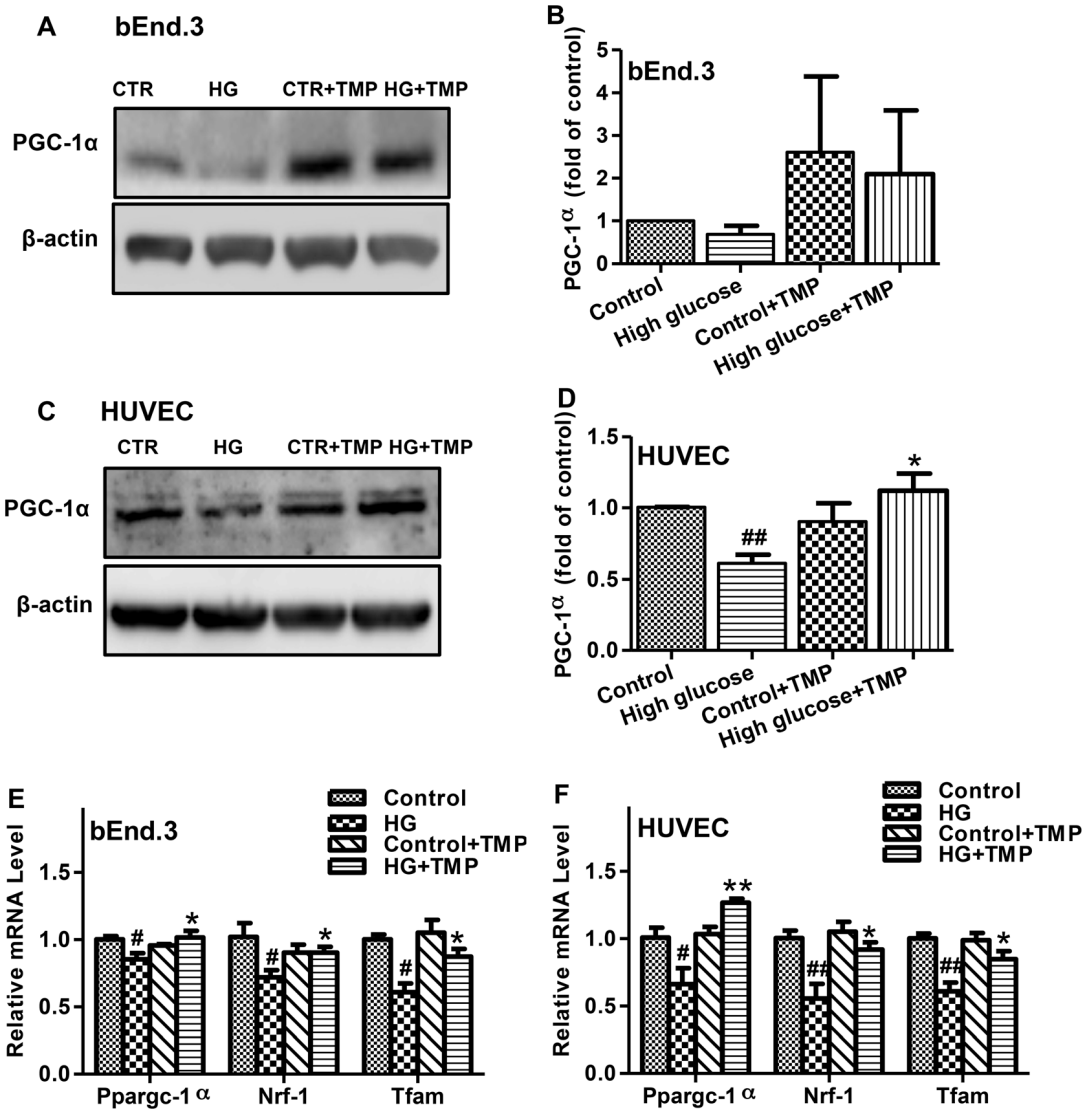
TMP up-regulated mitochondrial biogenesis-related factors. The bEnd.3 and HUVEC cells were treated with Control (5.6 mmol/L glucose, CTR), High glucose (30 mmol/L, HG), Control+ TMP (30 µmol/L) and High glucose+TMP (30 µmol/L) for 48 hrs. Levels of PGC-1α expression were analyzed by Western blotting in bEnd.3 (**A** and **B**) and HUVEC cells (**C** and **D**). Representative blots (**A** and **C**) and quantification data (**B** and **D**) are shown. Levels of PGC-1α, NRF-1 and TFAM mRNA were determined by quantitative RT-PCR in the treated bEnd.3 (**E**) and HUVEC (**F**). Data are mean ± SEM (n = 4). ^#^ *P*<0.05 vs. Control; ^##^ *P*<0.01 vs. Control; ^*^ *P*<0.05 vs. High glucose; ^**^ *P*<0.01 vs. High glucose.

PGC-1α plays an important role in governing the transcriptional control of mitochondrial biogenesis and respiratory function [18], [29]–[30]. In an attempt to elucidate the mechanism of TMP protection on mitochondrial function, the effects of TMP on PGC-1α expression were investigated. High glucose treatment significantly reduced PGC-1α expression in both bEnd.3 and HUVEC cells, whereas the high glucose-induced reduction of PGC-1α was completely reversed by the addition of TMP (Figure 6A–D). Correspondingly, high glucose treatment resulted in a significant reduction in mRNA levels of NRF-1 and TFAM, the two key downstream effectors of the PGC-1α pathway. In keeping with the changes in PGC-1α, TMP treatment completely prevented high glucose-induced reduction of NRF-1 and TFAM expression in bEnd.3 and HUVEC cells (Figure 6E–F). Serving as a control, TMP alone did not influence the mRNA levels of PGC-1α, NRF-1 and TFAM in both bEnd.3 and HUVEC cells under normal glucose conditions (Figure 6E–F). These results suggest a direct effect of TMP on mitochondrial biogenesis through up-regulation of PGC-1α, NRF-1 and TFAM expression.

### Effect of TMP on PGC-1α expression is dependent on SIRT1

**Figure 7:**
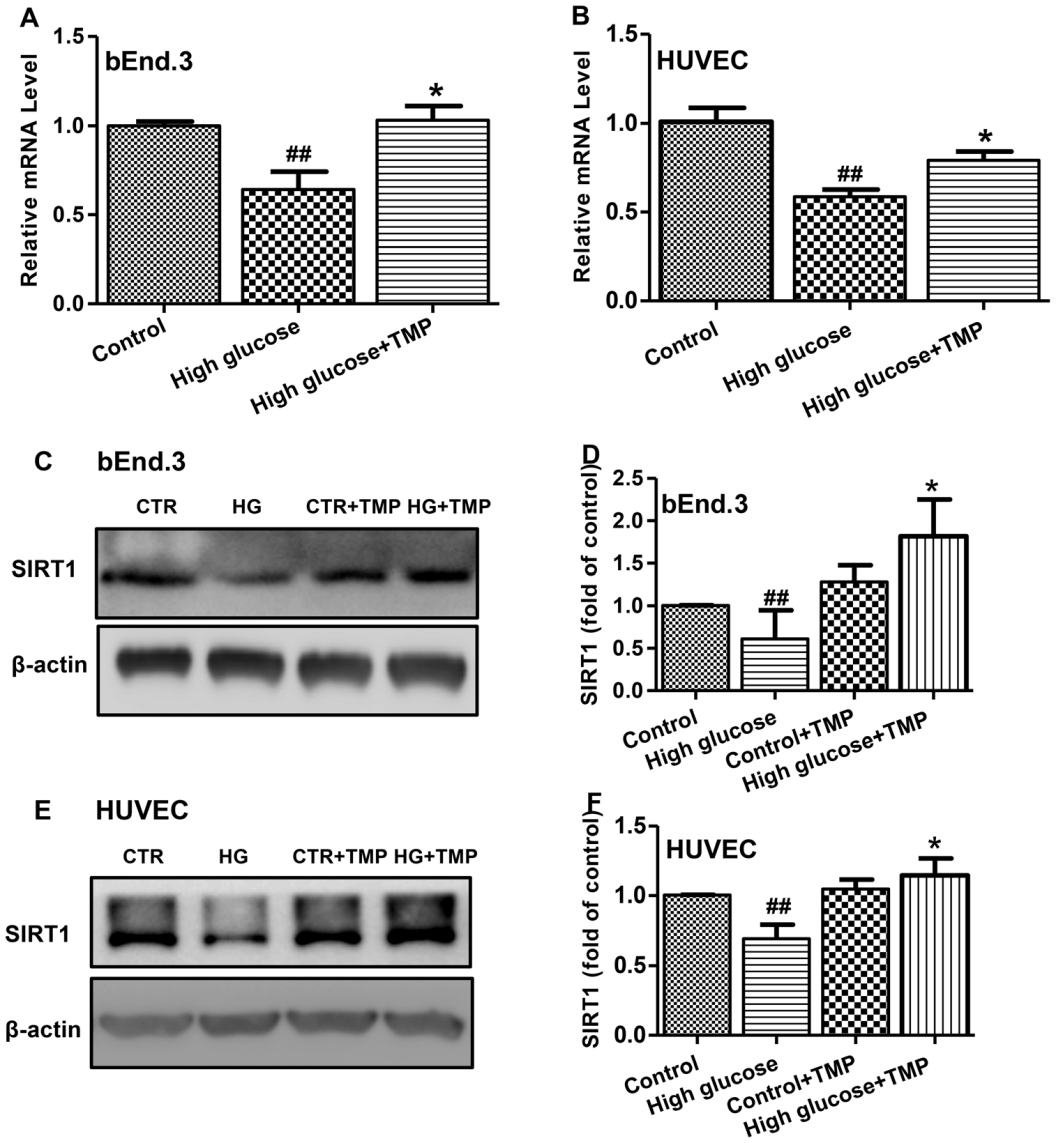
TMP up-regulated SIRT1 expression. The bEnd.3 and HUVEC cells were treated with Control (5.6 mmol/L glucose, CTR), High glucose (30 mmol/L, HG), Control+ TMP (30 µmol/L) and High glucose+TMP (30 µmol/L) for 48 hrs. The mRNA levels of SIRT1 were determined by RT-PCR (**A** and **B**). Levels of SIRT1 expression were analyzed by Western blotting in bEnd.3 (**C** and **D**) and HUVEC cells (**E** and **F**). Representative blots (**C** and **E**) and quantification data (**D** and **F**) are shown. Data are mean ± SEM (n = 4). ^#^ *P*<0.05 vs. Control; ^##^ *P*<0.01 vs. Control; ^*^ *P*<0.05 vs. High glucose; ^**^ *P*<0.01 vs. High glucose.

**Figure 8:**
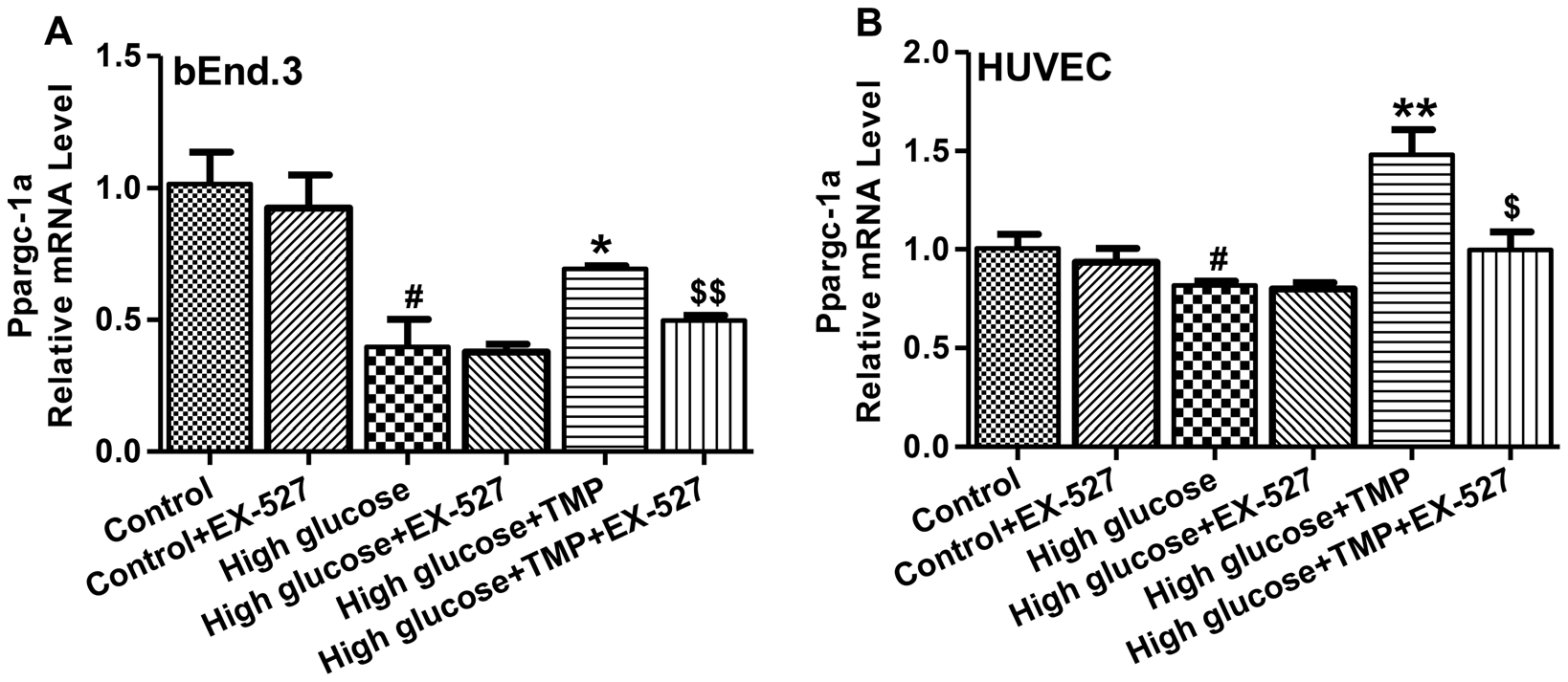
EX-527 abolished TMP-induced PGC-1α up-regulation. Levels of PGC-1α mRNA were determined by quantitative RT-PCR in the treated bEnd.3 (**A**) and HUVEC cells (**B**). Data are mean ± SEM (n = 4). ^#^ *P*<0.05 vs. Control; ^*^ *P*<0.05 vs. High glucose; ^$^ *P*<0.05 vs. High glucose +TMP; ^$$^ *P*<0.01 vs. High glucose +TMP.

Because SIRT1 is key regulator of PGC-1α expression through epigenetic regulation of the gene [19], we sought to investigate the effect of TMP on SIRT1. High glucose treatment resulted in a significant decrease in SIRT1 expression at both mRNA (Figure 7A–B) and protein levels (Figure 7C–F) in endothelial cells. Notably, TMP treatment resulted in a significant increase in SIRT1 expression and completely reversed high glucose-induced SIRT1 reduction in bEnd.3 and HUVEC cells (Figure 7A–F). To investigate whether TMP-induced up-regulation of PGC-1α is associated with SIRT1, we used EX-527, a specific inhibitor of SIRT1 [31]. As shown in Figure 8A–B, TMP-induced increases in PGC-1α expression was significantly attenuated by EX-527 in bEnd.3 and HUVEC cells exposed to high glucose. In contrast, EX-527 alone had no effect on PGC-1α expression in both bEnd.3 and HUVEC cells exposed to either normal or high glucose. Together, these data suggest a SIRT1-dependent effect of TMP on the PGC-1α expression in endothelial cells under high glucose conditions.

## Discussion

In this study, we report an ability of TMP to relax rat aortic rings in an endothelium-dependent manner. TMP was shown to increase NO generation in high glucose-treated endothelial cells, which may account for the relaxant effects on the endothelium-intact aortic rings. Moreover, TMP decreased mitochondrial superoxide anion production, up-regulated complex III expression and elevated mitochondrial membrane potential, all of these actions may contribute to the ameliorative effects of TMP on endothelial dysfunction. In addition, TMP was able to activate the PGC-1α-mediated pathway, suggesting that the protective effect of TMP on endothelial function is related to mitochondrial biogenesis. Interestingly, TMP increased the expression of SIRT1, and EX-527 abolished the TMP-induced up-regulation of PGC-1α, indicating SIRT1 could be a molecular target of TMP's action.

In our previous study, QHYH was demonstrated to reduce urinary albumin excretion (UAER) in type 2 diabetes patients [32]. As UAER is regarded as an important marker of systemic endothelial function [33], QHYH was suggested to have protective effects against endothelial dysfunction. This notion is now supported by the current findings that TMP profoundly relax endothelium-intact aortic rings (Fig 1). Furthermore, in keeping with our previous study [17], high glucose-induced NO reduction in endothelial cells was completely reversed by TMP treatment, indicating that TMP relaxes endothelium-intact aortic rings by enhancing NO production and that TMP improves high glucose-induced endothelial dysfunction.

Oxidative stress is an important mechanism underlying endothelial dysfunction, while mitochondria is a major source of ROS production [34]. The accumulation of ROS not only leads to mitochondrial dysfunction, but also induces entire cell damage when ROS are transformed to hydroxyl radicals and hydrogen peroxide [35]. Given the effect of TMP in improvement of high glucose-induced endothelial dysfunction, we thought TMP may function as an antioxidant in mitochondria. Indeed, TMP reduced total ROS generation in high glucose-treated endothelial cells (Fig 3). Furthermore, the antioxidant effects of TMP in mitochondria were confirmed by the MitoSOX™ assays, showing that TMP treatment reduced superoxide anion generation in endothelial cells exposed to high glucose (Fig 4). In addition, TMP up-regulated complex III protein levels and reversed mitochondrial membrane potential in high glucose-treated bEnd.3 and HUVEC cells (Fig 5), suggesting that TMP could alleviate mitochondrial dysfunction under high glucose-induced oxidative stress conditions.

PCG-1α is an important co-activator involved in the regulation of intracellular oxidative stress and mitochondrial biogenesis [30], [36]–[37]. Over-expression of PCG-1α in endothelial cells has been reported to up-regulate NRF-1 and TFAM, mitochondrial membrane potential and inhibit apoptosis [18], [38]–[39]. In this study, the effects of TMP on PCG-1α expression were evaluated by real-time PCR and western-blot assays. TMP enhanced PCG-1α expression in bEnd.3 and HUVEC cells treated with high-glucose (Fig 6A–D). In addition, TMP increased NRF-1 and TFAM mRNA expression in these endothelial cells (Fig 6E–F), implying TMP could enhance mitochondrial biogenesis by activation of the PCG-1α pathway.

SIRT1, a NAD^+^-dependent histone deacetylase, acts as a sensor to regulate intracellular oxidative stress status by deacetylation of its substrates, including PCG-1α [40]. SIRT1-mediated PCG-1α deacetylation is necessary for the activation of mitochondrial fatty acid oxidation genes [41]. Our study shows that TMP up-regulated SIRT1 mRNA and protein expression in bEnd.3 and HUVEC cells exposed to high glucose (Fig 7). EX-527 is a potent and specific small molecular inhibitor of SIRT1 [31]. After treatment with EX-527, TMP up-regulated PGC-1α expression was dramatically diminished (Fig 8). These data suggest that the effects of TMP in promoting mitochondrial biogenesis and amelioration of mitochondrial dysfunction are related to the up-regulation of SIRT1. Therefore, SIRT1 is likely to be a potential molecular target of TMP for amelioration of intracellular oxidative stress and endothelial function.

Collectively, TMP is demonstrated in this study to perform protective effects on endothelium at both organ and cell levels. TMP acts as an antioxidant in mitochondria and improves mitochondrial dysfunction, which contributes to its benefit effects on endothelium. In addition, the protective effects of TMP is likely associated with enhanced mitochondrial biogenesis through the SIRT1-dependent PGC-1α pathway, illustrating a new molecular mechanism underlying the antioxidant ability of TMP as observed in the clinic of TCM.
